# 4R-Cembranoid Improves Outcomes after 6-Hydroxydopamine Challenge in Both *In vitro* and *In vivo* Models of Parkinson's Disease

**DOI:** 10.3389/fnins.2017.00272

**Published:** 2017-05-29

**Authors:** Jing Hu, P. A. Ferchmin, Ann M. Hemmerle, Kim B. Seroogy, Vesna A. Eterovic, Jiukuan Hao

**Affiliations:** ^1^Division of Pharmaceutical Sciences, James L. Winkle College of Pharmacy, University of CincinnatiCincinnati, OH, United States; ^2^Department of Neurosciences, School of Medicine, Universidad Central del CaribeBayamón, Puerto Rico; ^3^Department of Neurology and Rehabilitation Medicine, University of CincinnatiCincinnati, OH, United States

**Keywords:** cembranoid, inflammation, neuroprotection, caspase-3, tyrosine hydroxylase, Parkinson's disease, 6-OHDA

## Abstract

(1S, 2E, 4R, 6R,-7E, 11E)-2, 7, 11-cembratriene-4, 6-diol (4R) is one of the cembranoids found in tobacco leaves. Previous studies have found that 4R protected acute rat hippocampal slices against neurotoxicity induced by N-methyl-D-aspartate (NMDA) and against the toxic organophosphorus compounds paraoxon and diisopropylfluorophosphate (DFP). Furthermore, *in vivo*, 4R reduced the infarct size in a rodent ischemic stroke model and neurodegeneration caused by DFP. The present study expanded our previous study by focusing on the effect of 4R in Parkinson's disease (PD) and elucidating its underlying mechanisms using 6-hydroxydopamine (6-OHDA)-induced injury models. We found that 4R exhibited significant neuroprotective activity in the rat unilateral 6-OHDA-induced PD model *in vivo*. The therapeutic effect was evident both at morphological and behavioral levels. 4R (6 and 12 mg/kg) treatments significantly improved outcomes of 6-OHDA-induced PD *in vivo* as indicated by reducing forelimb asymmetry scores and corner test scores 4 weeks after injection of 6-OHDA (*p* < 0.05). The therapeutic effect of 4R was also reflected by decreased depletion of tyrosine hydroxylase (TH) in the striatum and substantia nigra (SN) on the side injected with 6-OHDA. TH expression was 70.3 and 62.8% of the contralateral side in striatum and SN, respectively, after 6 mg/kg 4R treatment; furthermore, it was 80.1 and 79.3% after treatment with 12 mg/kg of 4R. In the control group, it was 51.9 and 23.6% of the contralateral striatum and SN (*p* < 0.05). Moreover, 4R also protected differentiated neuro-2a cells from 6-OHDA-induced cytotoxicity *in vitro*. The activation of p-AKT and HAX-1, and inhibition of caspase-3 and endothelial inflammation, were involved in 4R-mediated protection against 6-OHDA-induced injury. In conclusion, the present study indicates that 4R shows a therapeutic effect in the rat 6-OHDA-induced PD model *in vivo* and in 6-OHDA-challenged neuro-2a cells *in vitro*.

## Introduction

Parkinson's disease (PD) is the second most common neurodegenerative disease affecting more than one million people in the USA. It is estimated that 10 million people have PD worldwide (Foundation PsD., 2016). However, only a few pharmacological drugs are available for treating the symptoms of PD and none suppress the relentless progression of the disease. Some drugs like levodopa can lead to oxidative damage causing long-term side effects (Vijayakumar and Jankovic, 2016). Therefore, there is an urgent need for new therapeutics for PD.

Parkinson's disease is characterized by the progressive death of dopaminergic neurons in the pars compacta region of the substantia nigra (SN) and dopamine depletion in the striatum, which result in motor disability and postural abnormalities. Lewy bodies (LBs) and Lewy neurites (LNs) are the characteristics of pathological changes in PD. The LBs and LNs in PD are mainly composed of the aggregated form of a presynaptic protein, α-synuclein (α-Syn). α-Syn aggregation and mutations of α-Syn are associated with early-onset PD. In addition to the direct neurotoxicity of a-Syn, aggregation of the aberrant protein may also cause degeneration of dopaminergic neurons by inducing neuroinflammation (McGeer et al., 1988; Imamura et al., 2003). For instance, modification of the immune system in the a-Syn transduction PD model through knockout of proinflammatory-associated genes, such as MHCII, CX3CR1, or FccRIII, reduces M1/Th1 inflammatory reactions and protects dopaminergic neurons from degeneration (Harms et al., 2013). Although the pathophysiological mechanisms of PD remain poorly understood, it is well known that inflammation and apoptosis contribute to neurodegeneration in PD. Activation of microglia and release of inflammatory cytokines in the SN have been reported in PD patients (Langston et al., 1983; McGeer et al., 1988; Orr et al., 2002) and PD animal models (Akiyama and McGeer, 1989; Gao et al., 2002, 2003; McGeer et al., 2003). Endothelial inflammation and dysfunction were observed at the blood-brain barrier (BBB) in PD as well (Grammas et al., 2011). Inflammation at the level of the brain endothelium plays a role in the pathology of PD by triggering the release of various inflammatory cytokines, promoting recruitment of activated lymphocytes and monocytes into the brain, and activating microglia and other glial cells (Danton and Dietrich, 2003). Up-regulation of inflammatory mediators at the BBB is closely related to activation of nuclear factor kappa B (NF-κB), a master switch of inflammation (Baldwin, 2001; Keifer et al., 2001). Normally, NF-κB is inactive by association with the inhibitor of NF-κB protein (IκB) in the cytosol (Jacobs and Harrison, 1998; Schwaninger et al., 2006). Upon activation, NF-κB is disassociated from IκB and is translocated into the nucleus, which leads to rapid up-regulation or down-regulation of its target gene expression. For example, elevated levels of endotoxin and proinflammatory cytokines increase NF-κB activity leading to up-regulation of a variety of inflammatory mediators in brain endothelial cells, such as interleukin-1 (Quan et al., 1998a), tumor necrosis factor (TNF) (Nadeau and Rivest, 1999; Yang et al., 1999), cyclooxygenase-2 (Quan et al., 1998b), inducible nitric oxide synthase (Wong et al., 1996), and the adhesion molecules intercellular adhesion molecule 1 (ICAM-1) and vascular cell adhesion molecule 1 (VCAM-1) (Lindsberg et al., 1996; Henninger et al., 1997; Stanimirovic et al., 1997). The inflammation of the BBB leads to neuronal injury and death (de Vries et al., 1996), which may impact midbrain dopaminergic neurons, since these cells are more vulnerable to various insults (Gonzalez-Hernandez et al., 2010). Therefore, activation of NF-κB at the BBB is implicated in the development of PD (Hunot et al., 1997; Togo et al., 2001). It has been reported that NF-κB is also activated in dopaminergic neurons of PD patients and rodents administered the catecholaminergic neurotoxin 6-hydroxydopamine (6-OHDA) (Hunot et al., 1997; Levites et al., 2002). These observations suggest that chronic inflammation may induce progressive neurodegeneration in the nigrostriatal system (McGeer et al., 2001; McGeer and McGeer, 2004).

There are also other mechanisms that contribute to neurodegeneration by triggering dopaminergic neuronal apoptosis in PD. One well-known mechanism is through inhibition of Akt, an anti-apoptotic protein, which plays an important role in neuroprotection. The Akt molecule is also called protein kinase B (PKB) with a structure similar to protein kinase A (PKA) (Staal, 1987; Cheng et al., 2005). Akt is the downstream kinase of phosphoinositide-3-kinase (PI3K). Over decades, the Akt pathway has been proven as an anti-apoptotic pathway, which is involved in cell survival and growth. As a survival kinase, Akt is activated by PI3K through phosphorylation. Recruitment of cytosolic Akt to the cell membrane via by phosphatidylinositol 1,3,5 triphosphate (PIP3) facilitates its phosphorylation and activation leading to neuronal survival (Namikawa et al., 2000; Alvarez-Tejado et al., 2001; Bijur and Jope, 2003; Leeds et al., 2005; Zhong et al., 2005). The p-Akt phosphorylates a number of apoptosis-regulatory molecules such as BAD, caspase 3 and 9, GSK-3β, IκB kinase, cAMP-responsive element binding protein (CREB), forkheads, and proline-rich Akt substrate (PRAS). Apoptotic functions of BAD, caspase 3 and 9, GSK-3β, forkheads, and PRAS are inhibited, whereas anti-apoptotic functions of CREB and Bcl-2 are activated by p-Akt (Chan, 2004; Harada et al., 2004; Fernandez-Gomez et al., 2006). Akt also works as a regulator to regulate cerebral blood flow by phosphorylation of eNOS in endothelial cells. The increased eNOS activity by Akt leads to an increase in cerebral blood flow (Lin et al., 2010). In addition to neuroprotection, Akt also plays an important role in neurogenesis. Neuronal differentiation and neurite outgrowth are essential during development of the nervous system and are crucial in neurogenesis. Akt has consistently been shown to have a positive influence on neuronal differentiation, neurite outgrowth, and neurite elongation in primary neurons (Kimura et al., 1994; Namikawa et al., 2000; Nakagomi et al., 2003; Tornieri et al., 2006; Tucker et al., 2006, 2008; Lim and Walikonis, 2008; Zheng et al., 2008). It has been shown that neurotrophins, such as nerve growth factor (NGF), brain-derived neurotrophic factor (BDNF) and neurotrophin-3 (NT3) promote neurogenesis by binding to Trk receptor tyrosine kinases, which then activate PI3K-Akt signal transduction (Huang and Reichardt, 2003). Importantly, research has shown that upregulation of Akt has a neuroprotective effect in PD (Hashimoto et al., 2004; Ries et al., 2006; Wu et al., 2007; Quesada et al., 2008; Yasuda et al., 2011). Therefore, upregulation of Akt could potentially be used alone or in combination with other therapeutic strategies to treat neurodegeneration in PD.

Another protein, hematopoietic lineage substrate-1–associated protein X-1 (HAX-1), recently was found to have an anti-apoptotic function. HAX-1 has a structure similar to Bcl-2 and is expressed in various tissues including brain and peripheral leukocytes (Hippe et al., 2006; Carlsson et al., 2008; Trebinska et al., 2010). HAX-1 interacts with a number of molecules, such as caspase-9 (Han et al., 2006), caspase-3 (Rami and Langhagen, 2012), heat-shock protein 90 (Lam et al., 2013), and mitochondrial proteases (Chao et al., 2008; Han et al., 2010), to regulate cell survival and growth. For instance, interactions between HAX-1 and mitochondrial proteases, including presenilins-associated rhomboid-like and high temperature-regulated A2 also known as Omi, are required to suppress cell apoptosis (Chao et al., 2008). A deficit of HAX-1 is associated with neuronal apoptosis and lymphocyte infiltration in the brain (Chao et al., 2008). Recent evidence indicates that HAX-1 is also involved in the development of the nervous system and pathologies of neurological diseases, such as ischemic stroke (Hao et al., 2008; Rami and Langhagen, 2012), brain trauma (Hao et al., 2008; Shi et al., 2011), and autosomal recessive severe congenital neutropenia (Klein et al., 2007; Rezaei et al., 2007; Carlsson et al., 2008; Ishikawa et al., 2008). Importantly, HAX-1-null mice have neurodegenerative parkinsonian features (Chao et al., 2008), which may indicate the involvement of HAX-1 deficit in the pathology of PD.

The present study focuses on the effect of 4R on PD and elucidating its underlying mechanisms using 6-OHDA-induced PD models *in vitro* and *in vivo* and a TNF-α-induced inflammatory model *in vitro*. This study was designed to (i) evaluate the effect of 4R in the setting of 6-OHDA-induced experimental PD in the rat at the morphological and functional levels and (ii) elucidate its underlying mechanisms using *in vitro* PD and *in vitro* inflammatory models.

## Methods

### *In vitro* PD model

Neuro-2a cells, a mouse neural crest-derived cell line, purchased from ATCC (Manassas, VA, USA), were grown in DMEM with high glucose and L-glutamine (Hyclone, Logan, UT, USA) supplemented with 10% (v/v) heat-inactivated fetal bovine serum (FBS) (Atlanta Bio Inc. GA, USA), and 1% (v/v) 10,000 IU/ml penicillin/10,000 mg/ml streptomycin (ATCC). Neuro-2a cells differentiate into dopaminergic cells in the presence of dibutyryl cyclic adenosine monophosphate (dbcAMP) and reduced serum. Briefly, cells were treated with dbcAMP at a final concentration of 2.5 mM in the cell culture medium with 0.5% FBS for 72 h (Tremblay et al., 2010). Then, the cells were incubated with a 6-OHDA-ascorbic acid solution (8, 12.5, or 25 μM) (Sigma-Aldrich Co. LLC., St. Louis, MO, USA) for 24 h to induce the neurotoxicity considered to be an *in vitro* PD model (Ochu et al., 1998; Storch et al., 2000). 4R (10, 20, or 50 nM) was added at the same time as 6-OHDA and incubated for 24 h. 4R was prepared by Dr. K. El Sayed (School of Pharmacy, University of Louisiana, Monroe, LA) (El Sayed et al., 2008).

### *In vitro* inflammatory model

The brain endothelial cell line (bEND5 cells) derived from mouse brain and immortalized with polyoma middle T oncogene was a generous gift from Dr. Ulrich Bickel, Texas Tech University. bEND5 cells were grown in DMEM media (Hyclone) supplemented with 10% (v/v) FBS, 1 mM sodium pyruvate, 4 mM L-glutamine, 1% (v/v) non-essential amino acids, 1% (v/v) 10,000 IU/ml penicillin/10,000 mg/ml streptomycin (all from ATCC). The inflammatory model was developed by stimulating bEND5 cells with 50 ng/ml TNF-α overnight (Tomita et al., 1998) and then treating with 4, 8, or 16 μM 4R for 24 h.

### Western blot

Cells were lysed in ice-cold 1X RIPA lysis buffer (Santa Cruz Biotechnology, Inc., CA, USA) containing: PMSF, sodium orthovanadate and protease inhibitor cocktail. After incubation in ice-cold buffer for 30 min, the cell lysate was centrifuged at 10,000 × g for 10 min at 4°C, and the supernatant was harvested. The detailed methods of the Western blot procedure were performed as previously described (Gao et al., 2003). The intensity of the chemiluminescence signal was normalized to that of β-actin as indicated. The following antibodies and the respective dilutions were used: 1:2,000 for β-actin, 1:2,000 for VCAM-1, 1:1,000 for cleaved caspase-3 and 1:2,000 for anti-rabbit secondary antibody (Cell Signaling Inc., Danvers, MA, USA); 1:1,000 for tyrosine hydroxylase (TH), 1:1,000 for p-Akt, 1:1,000 for ICAM-1 and 1:2,000 for anti-goat secondary antibody (Santa Cruz Biotechnology Inc.). The TATA box binding protein (TBP) was the nuclear protein loading control for p65. The antibody for TBP was from ABCAM and was used at 1:1,000 dilution.

### Adhesion assay

U937 cells (a monocyte cell line from ATCC) were cultured in RPMI 1640 medium (Hyclone) supplemented with 10% FBS, 2 mM l-glutamine and 1% (v/v) 10,000 IU/ml penicillin /10,000 mg/ml streptomycin (ATCC). All of the cell lines were maintained at 37°C, 5% CO_2_, and 95% relative humidity. U937 cells were labeled with 5 mg/mL BCECF-AM (2N,7N-bis-(2-carboxyethyl)-5-(and-6)-carboxyfluorescein), (Sigma Inc.) for 30 min at 37°C, washed and re-suspended in serum-free media. bEND5 cells were cultured and incubated with 4R in a 24-well plate for 8 h followed by 16 h of TNF-α (50 ng/ml) stimulation, and then the cells were co-cultured with BCECF-AM-labeled U937 cells (10^6^ cells/well) for 30 min at 37°C. Non-adhering U937 cells were removed, and cells were washed with phosphate-buffered saline (PBS), and then lysed in 0.1% Triton X-100 in 0.1 M Tris-HCl (pH 7.4) (Sigma-Aldrich). Fluorescence (F) was measured with a microplate fluorescence reader (POLAR star OPTIMA, BMG Labtechnologies, Ortenberg, Germany) using excitation at 492 nm and emission at 535 nm. The monocyte adhesion was calculated as Adhesion (%) = 100 × F_sample_/F _total_ (fluorescence intensity of 10^6^cells). The blank control group is the bEND5 cells without incubation of TNF-alpha.

### MTT assay

Neuro-2a cells were cultured in a 96-well plate in serum-reduced medium with 2.5 mM dbcAMP for 72 h to generate differentiated neuro-2a cells, dopaminergic-like cells. The differentiated neuro-2a cells were co-incubated with different concentrations of 6-OHDA (8, 12.5, and 25 μM) and either 4R (10, 20, and 50 nM) or DMSO (vehicle) for 24 h. The protective effect of 4R on cell viability was measured by the 3-(4, 5-dimethylthiazol-2-yl)-2, 5-diphenyltetrazolium bromide (MTT) assay. Briefly, MTT (Sigma-Aldrich) solution (5 mg/ml) was added 1:10 to the cell culture medium at 24 h after incubation with 4R or DMSO, and incubated for 2 h at 37°C. Next, the medium was removed, and 200 μl DMSO was added. The absorbance of the reaction product was measured in a plate reader at 570 and 690 nm. OD background was subtracted from the 570 nm OD total signal.

### *In vivo* 6-OHDA PD model

All animal procedures were approved by the Institutional Animal Care and Use Committee at the University of Cincinnati and complied with pertinent NIH guidelines for care and use of animals. Sprague Dawley rats (body weight 200-300 g) supplied by Charles River (Wilmington, MA, USA) were kept under standardized light/dark (12 h), temperature (22°C) and humidity (70%) conditions, with rodent chow and water available. Rats were anesthetized with 100 mg/kg ketamine and 20 mg/kg xylazine (Butler Animal Health Supply Inc.) and positioned in a stereotaxic frame. To achieve a unilateral lesion of the nigrostriatal pathway, 6-OHDA was injected into two sites of the right striatum: AP: +1.6 mm; ML: −2.4 mm; DV: −4.2 mm; AP: +0.2 mm; ML: −2.6 mm; DV:−7.0 mm (Paxinos and Watson, 2007). The solution of 6-OHDA (3 μg/μl) was prepared in a 0.2% ascorbic acid saline. At each site, 2 μl of 6-OHDA or vehicle was injected at a rate of 0.2 μl/min for 10 min, with the needle left in place for 5 min before and after the injection. The final dosage of 6-OHDA was 12 μg per rat. 4R subcutaneous administration (6 or 12 mg/kg) was started simultaneously with the striatal injection of 6-OHDA. Thereafter, 4R was administered every day for the first 3 days and then every other day for 4 weeks.

### Behavioral tests

The cylinder test was performed as previously described (Schallert et al., 2000; Hua et al., 2002; Hemmerle et al., 2014) to assess forelimb asymmetry every week after surgeries for 4 weeks. The rats were placed in an upright transparent cylinder, which encouraged vertical exploration of the walls using their forelimbs for weight support. The number of wall contacts made by each forelimb independent of the other or by both paws was scored for the calculation of limb usage asymmetry. Briefly, the asymmetry score was determined by the formula [(contralateral side + ½ both)/(ipsilateral side + contralateral side + both)]. A ratio of 0.5 suggests equal usage of both limbs, whereas scores less than 0.5 indicate motor deficits in the contralateral limb.

The corner test was performed as described previously (Hao et al., 2008). The rat was placed between two boards at a 30° angle facing the corner. Both sides of the vibrissae were stimulated when the rat reached deep into the corner, wherein the rat reared and turned either to the left or right to exit the corner. The 6-OHDA-treated rats preferentially turn toward the non-impaired (right) side. Turns involving a rearing movement were scored. A total of 10 proper turns was recorded for each animal in each session. After baseline evaluation before surgery, the corner test was performed once per week for 4 weeks after injection of 6-OHDA. The fraction of right turns out of the total number of turns served as the response variable.

### Immunohistochemistry and stereology

Rats were deeply anesthetized with a lethal dose of ketamine/xylazine and perfused intracardially with saline followed by 4% paraformaldehyde (PFA) (Sigma-Aldrich). Next, brains were dissected, post-fixed in PFA for 24 h and then placed in a 30% sucrose solution until they sunk. Brains were coronally sectioned at the striatal and ventral midbrain levels at 50-μm thickness using a sliding microtome. For immunohistochemistry, free-floating brain sections were washed three times with phosphate buffer and incubated with 0.3% H_2_O_2_ for 10 min. Next, the sections were treated with blocking buffer for 1 h and then incubated with TH primary antibody (Abcam; 1:200 dilution) in blocking buffer overnight in a humidified box at 4°C. After washing with phosphate buffer, the sections were incubated with HRP-conjugated anti-rabbit secondary antibody for 1 h and stained with DAB kit reagents (Thermo Fisher Scientific). Four brain sections per animal were analyzed for TH immunostaining in STR and SN, and the number of TH-positive cells was determined via unbiased stereological counting using Stereo Investigator (version 10.51) (West, 1993; Hemmerle et al., 2014). The contours were drawn at 2.5X and cell counting was performed at 60X using the optical fractionator. The sample sites were on a grid size of 170 × 100 for TH immunostaining with a guard zone set at 2.0 μm. The Gundersen correction was used to calculate the coefficient of error and was set at 0.10 or lower. The average absolute number of cells counted in the unlesioned (contralateral) SN was 800 cells/section. The optical densities of TH immunostaining in the striatum were determined via densitometric analysis using Scion Image (NIH).

### Statistical analysis

The outcomes of the 4R-treated animals exposed to the PD model *in vivo* were compared to that of animals treated with vehicle (DMSO) and a sham lesion. Statistical significance was determined by one-way ANOVA followed by the Holm-Sidak test using Sigma plot version 12.5 from Systat Software, Inc.

## Results

### 4R improves behavioral and morphological outcomes of 6-OHDA-induced nigrostriatal degeneration

**Figure 1:**
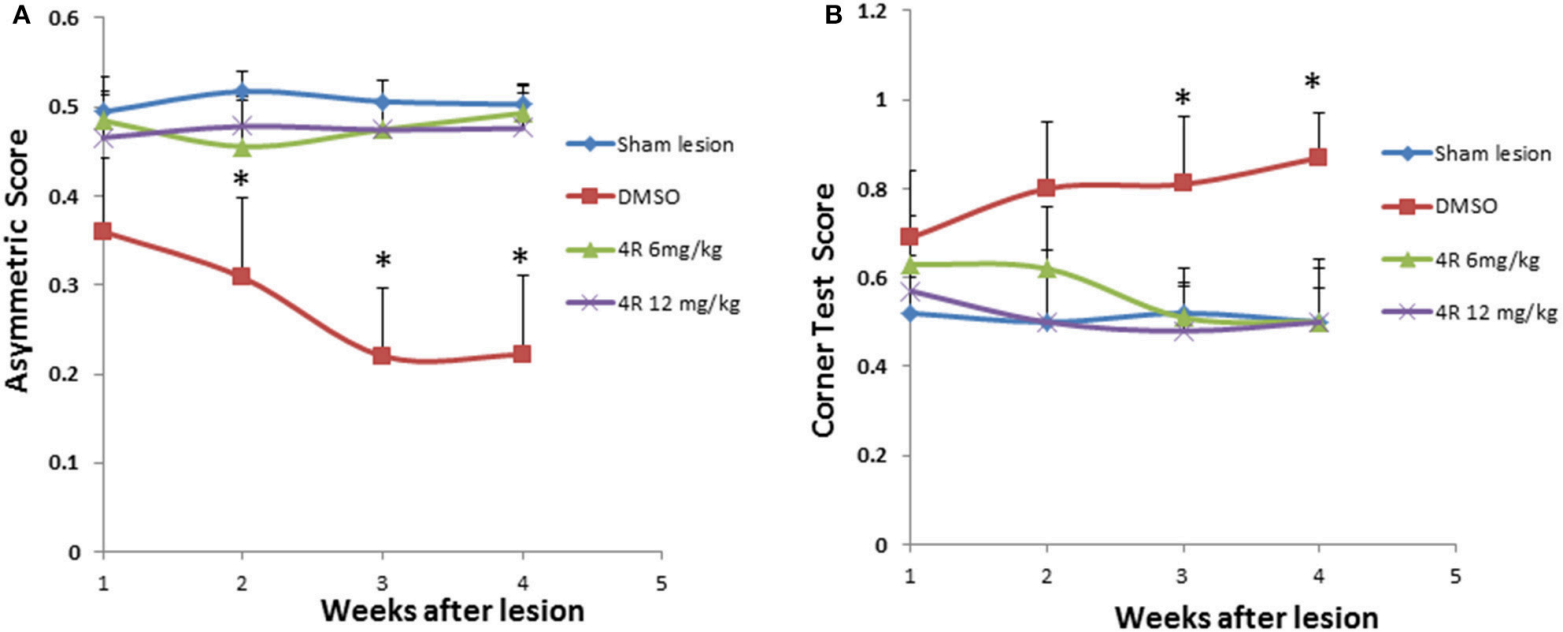
**The effect of 4R on the behavioral deficits of rats injected with 6-OHDA: (A)** Asymmetry scores from the cylinder test, Mean ± SD, *n* = 7, ^*^p < 0.05; **(B)** Corner test scores. Mean ± SD, *n* = 7, ^*^p < 0.05. The sham lesion group was subjected to a sham surgical operation without injections into the striatum. DMSO is the vehicle control, in which DMSO was injected into the animals via subcutaneous injection. There are two 4R treatment groups, 4R 6 mg/kg and 4R 12 mg/kg. The statistical analysis: ANOVA followed by the Holm-Sidak test.

The scores for the cylinder test (forelimb asymmetry) and corner test for a normal rat should be 0.5. If an asymmetry score is less than 0.5 or a corner test score is more than 0.5, it indicates unilateral motor functional deficiency. The results showed that asymmetry scores were significantly lower than 0.5, and the corner test scores were significantly higher than 0.5, after injection of 6-OHDA (pink curve in Figures 1A,B). The sham lesion did not have any effect on the scores of the behavioral tests (blue curve in Figure 1). However, treatment with both doses of 4R (6 mg/kg, 12 mg/kg) significantly improved outcomes in the behavior tests: asymmetry scores and corner test scores were close to 0.5 during all 4 weeks after injection of 6-OHDA in animals co-treated with 4R (Figure 1).

As shown in Figure 1A, the asymmetry scores were 0.48 ± 0.05, 0.46 ± 0.05, 0.47 ± 0.03, and 0.49 ± 0.03 in week-1, week-2, week-3, and week-4 after injection of 6 mg/kg 4R, respectively; they were 0.47 ± 0.05, 0.48 ± 0.03, 0.47 ± 0.03, and 0.48 ± 0.04 in week-1, week-2, week-3, and week-4 after injection of 12 mg/kg 4R, respectively. As shown in Figure 1B, the corner test scores were 0.63 ± 0.11, 0.62 ± 0.14, 0.51 ± 0.08, and 0.50 ± 0.14 in week-1, week-2, week-3, and week-4 after injection of 6 mg/kg 4R, respectively; they were 0.57 ± 0.08, 0.50 ± 0.12, 0.48 ± 0.14, and 0.50 ± 0.12 in week-1, week-2, week-3, and week-4 after injection of 12 mg/kg 4R, respectively.

**Figure 2:**
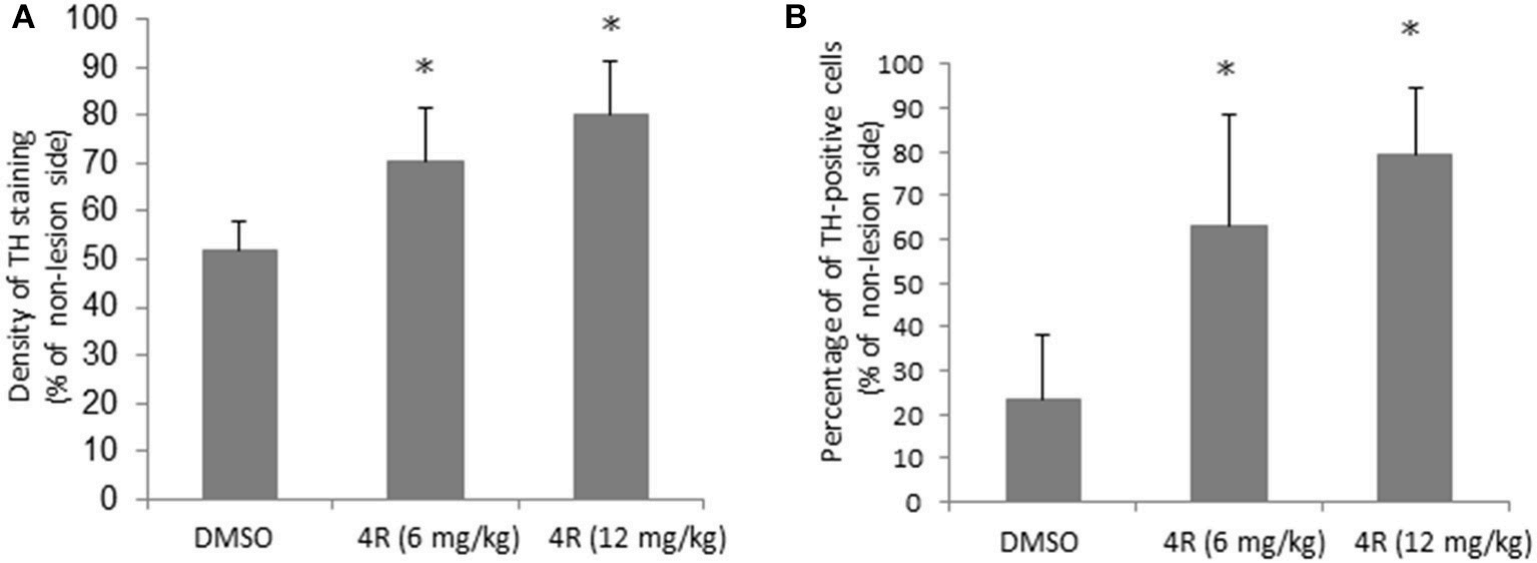
**The effect of 4R in TH immunostaining of the 6-OHDA-lesioned rats. (A)** Quantification of the effect of 4R treatment on TH immunostaining in the striatum. **(B)** Quantification of the effect of 4R treatment on TH immunostaining in the SN. Mean ± SD, *n* = 7, ^*^p < 0.05. The statistical analysis: ANOVA followed by the Holm-Sidak test.

**Figure 3:**
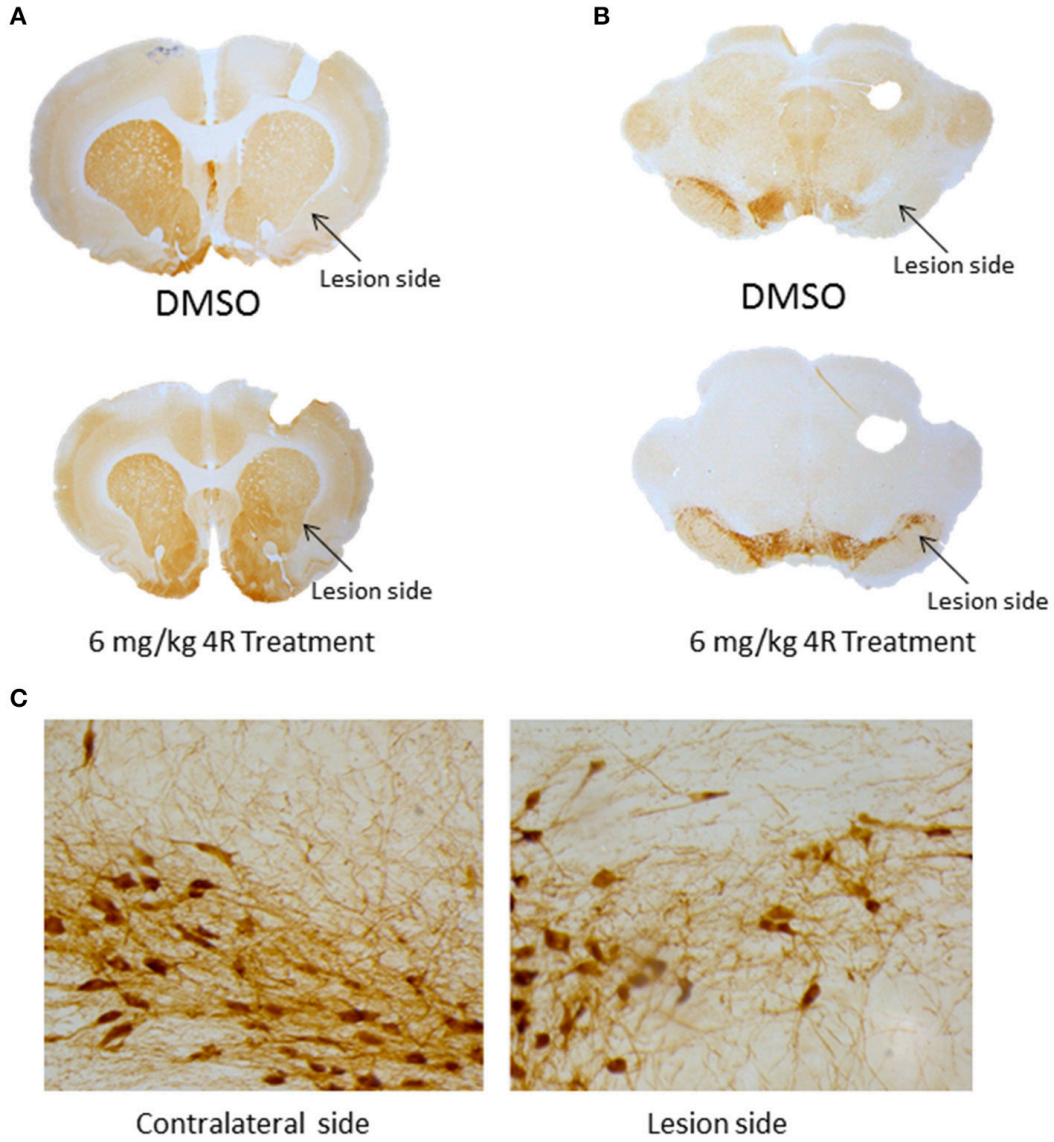
**(A)** Images of TH immunostaining in the striatum. **(B)** Images of TH immunostaining in the SN. **(C)** High magnification mages of TH immunostaining in the SN of contralateral side (left) and of lesion side (right) of 6 mg/kg-treated animal.

Furthermore, immunohistochemical evidence showed that the observed unilateral motor deficit was associated with 6-OHDA-induced TH depletion in the nigrostriatal pathway. Compared to the contralateral side, there was a significant depletion of TH-positive fibers and cells on the lesion side in the right striatum and right SN, respectively, after injection of 6-OHDA. The TH density and cell counts were 51.9 ± 5.7% (Figure 2A) and 23.6 ± 14.7% (Figure 2B) of the contralateral side in the right striatum and right SN, respectively, in the DMSO vehicle group. However, both doses of 4R treatment significantly attenuated TH depletion in both the striatum and SN of the lesioned side. TH expression was 70.3 ± 11.3% (Figure 2A) and 62.8 ± 25.8% (Figure 2B) of the contralateral side in the striatum and SN, respectively, after 6 mg/kg 4R treatment; TH expression was 80.1 ± 11% (Figure 2A) and 79.3 ± 15% (Figure 2B) of the contralateral side in the striatum and SN, respectively, after 12 mg/kg 4R treatment (Figure 2B). Representative images of TH immunostaining are illustrated in Figure 3.

### 4R protects differentiated neuro-2a cells from 6-OHDA-induced cytotoxicity *In vitro*

**Figure 4:**
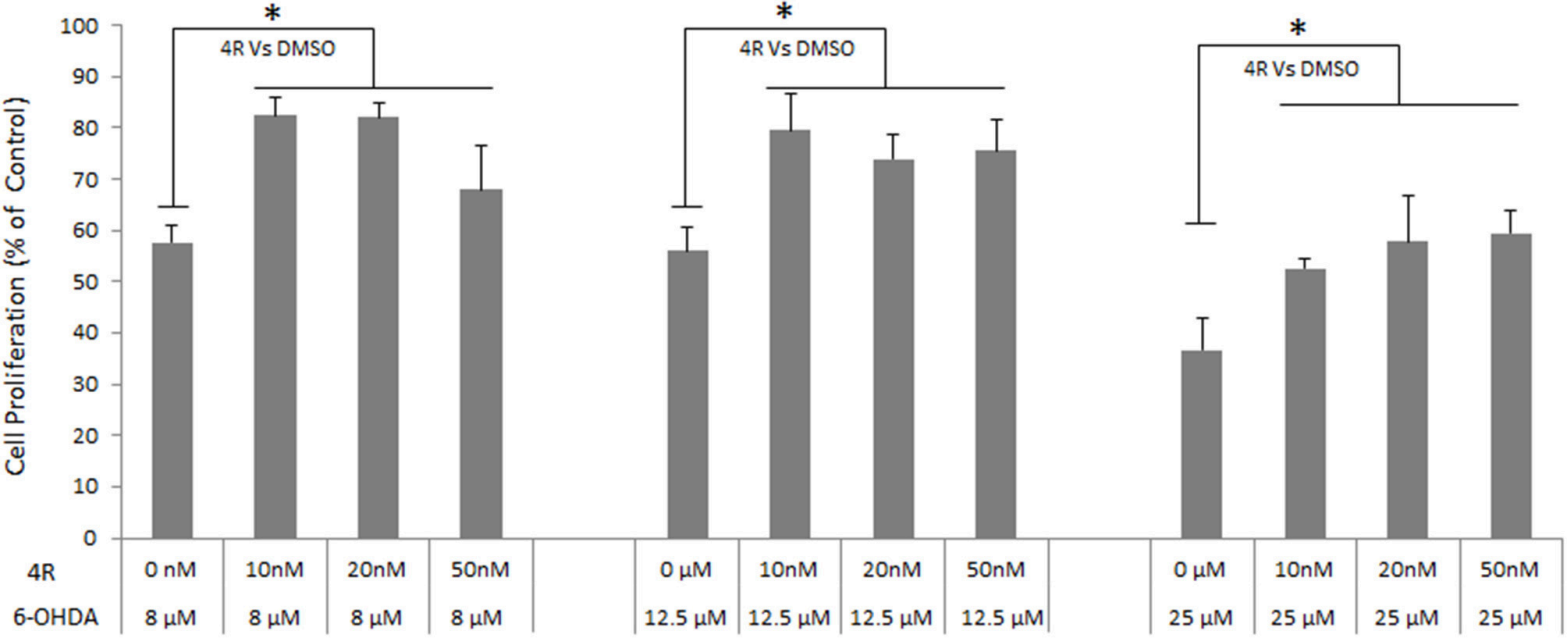
**The effect of different concentrations of 4R on the viability of differentiated neuro-2a cells in the ***in vitro*** PD model induced by several concentrations of 6-OHDA**. Mean ± SD, *n* = 6, ^*^p < 0.05.

**Figure 5:**
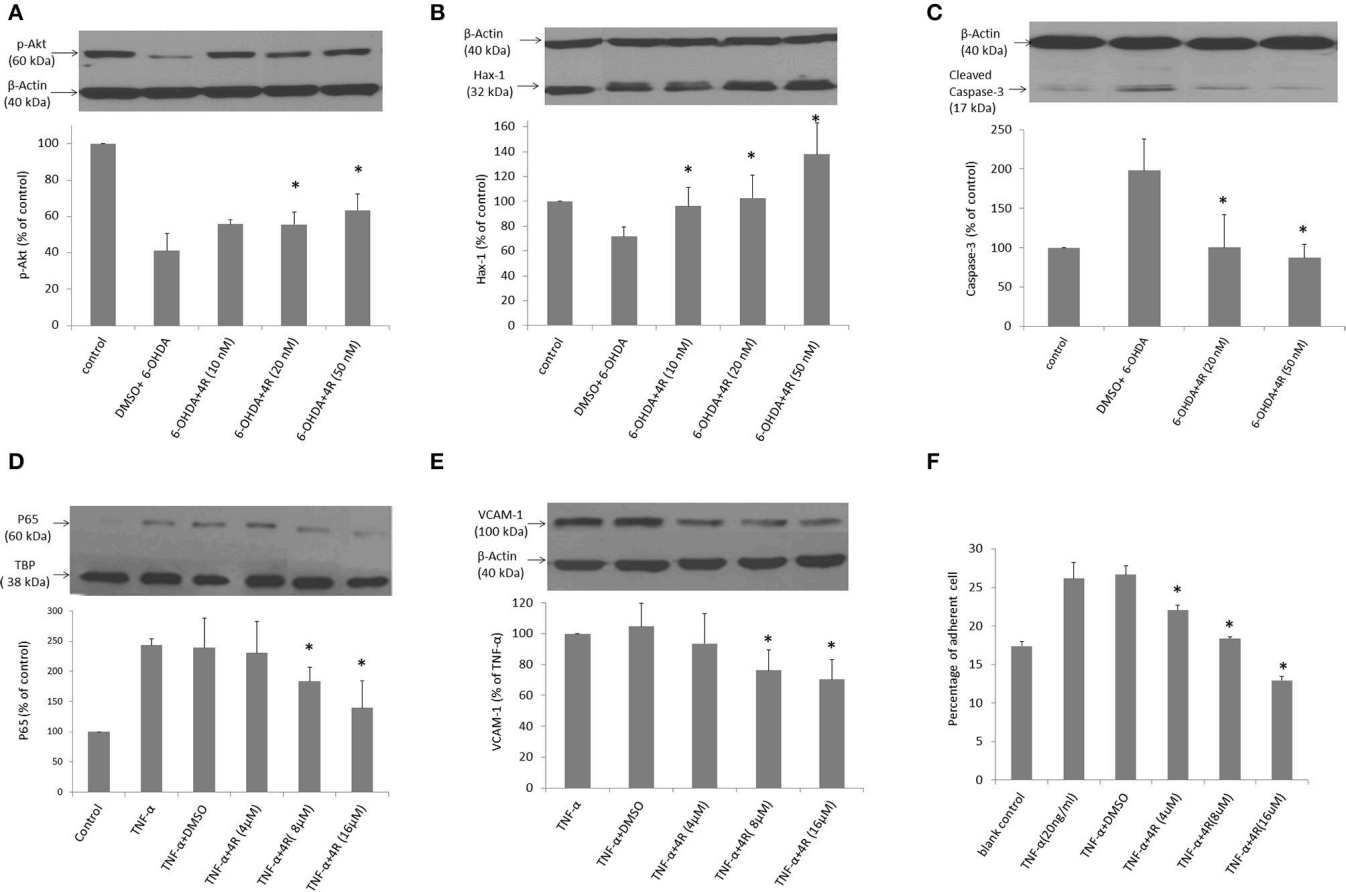
**(A–C)** The effect of 4R on expression of proteins of interest in the differentiated neuro-2a cells challenged with 6-OHDA: **(A)** p-Akt, Mean ± SD, *n* = 4–6, ^*^p < 0.05, 6-OHDA + 4R 20 nM vs. DMSO + 6-OHDA; 6-OHDA + 4R 50 nM vs. DMSO + 6-OHDA. **(B)** HAX-1, Mean ± SD, *n* = 4–6, ^*^p < 0.05, 6-OHDA + 4R 10 nM vs. DMSO + 6-OHDA; 6-OHDA + 4R 20 nM vs. DMSO + 6-OHDA; 6-OHDA + 4R 50 nM vs. DMSO + 6-OHDA. **(C)** cleaved caspase-3, Mean ± SD, *n* = 5, ^*^p < 0.05, 6-OHDA + 4R 20 nM vs. DMSO + 6-OHDA; 6-OHDA + 4R 50 nM vs. DMSO + 6-OHDA. **(D–E)** The effect of 4R on expression of proteins of interest in bEND5 cells challenged by TNF-α: **(D)** nuclear p65, Mean ± SD, *n* = 3–5, ^*^p < 0.05, TNF-α + DMSO vs. TNF-α + 4R 8 μM; TNF-α + DMSO vs. TNF-α + 4R 16 μM. TBP was used as housekeeping protein for p65. **(E)** VCAM-1, Mean ± SD, *n* = 3–4, ^*^p < 0.05, TNF-α + DMSO vs. TNF-α + 4R 8 μM; TNF-α + DMSO vs. TNF-α + 4R 16 μM. **(F)** The effect of 4R on monocyte adhesion to bEND5 cells in the TNF-α inflammatory model. Mean ± SD, *n* = 3, ^*^p < 0.05, TNF-α + DMSO vs. TNF-α + 4R 4 μM; TNF-α + DMSO vs. TNF-α + 4R 8 μM; TNF-α + DMSO vs. TNF-α + 4R 16 μM. The blank control group: the bEND5 cells were not exposed to TNF-α.

The viability of neuro-2a cells was decreased by 6-OHDA in a dose-dependent manner. Viability was reduced to 57.7 ± 3.1, 55.9 ± 4.7, and 36.7 ± 6.2% of controls after incubation with 8, 12.5, and 25 6-OHDA for 24 h, respectively (Figure 4). Three doses of 4R (10, 20, and 50 nM) treatment significantly improved cell viability in all 6-OHDA-challenged groups. The cell viability was 82.5 ± 3.5, 82.1 ± 2.8, and 67.9 ± 8.8% of control in 8 μM 6-OHDA groups after 10, 20, and 50 nM 4R treatment, respectively. The cell viability was 79.6 ± 7.1, 74.0 ± 4.6, and 75.6 ± 6.1% of control in 12.5 μM 6-OHDA groups after 10, 20, and 50 nM 4R treatment, respectively. The cell viability was 52.6 ± 2.0, 57.8 ± 9.0, and 59.5 ± 4.5% of control in 25 μM 6-OHDA groups after 10, 20, and 50 nM 4R treatment, respectively (Figure 4, *p* < 0.05). The cellular signaling pathways involved in 4R protection were investigated. We found that anti-apoptotic proteins such as p-AKT, HAX-1, and the apoptotic protein caspase-3, were involved in 4R-mediated protection against 6-OHDA-induced injury. 25 μM 6-OHDA significantly decreased phosphorylation of Akt and the expression of HAX-1 in differentiated neuro-2a cells (Figures 5A,B). Conversely, there was an increase of cleaved caspase-3 levels after incubation with 6-OHDA in the same samples (Figure 5C). Furthermore, 4R significantly increased the level of p-Akt (Figure 5A) and restored HAX-1 to base levels (Figure 5B). Simultaneously, 4R decreased the level of cleaved caspase-3 induced by 6-OHDA (Figure 5C).

### Effect of 4R on endothelial inflammation induced by TNF-α in murine brain-derived endothelial cells (bEND5 cells)

We studied the anti-inflammatory effect of 4R on TNF-α-induced inflammation in brain-derived endothelial cells. TNF-α upregulated the expression of the nuclear NF-κB subunit, p65, and the NF-κB-dependent inflammatory cytokine, VCAM-1, in the bEND5 cells. 4R attenuated the increased level of p65 and VCAM-1 induced by TNF-α (Figures 5D,E). TNF-α dramatically increased the level of p65 to 243 ± 10% of controls. Treatment with 4R significantly reduced the level of p65 to 230 ± 52, 183 ± 24, and 138 ± 44% at concentrations of 4, 8, and 16 μM, respectively, suggesting that 4R blocked the nuclear translocation of NF-κB in a dose-dependent manner (Figure 5D). VCAM-1 was expressed at a very low level under normal conditions, but TNF-α strongly induced its expression. However, the expression of VCAM-1 was significantly reduced to 93 ± 19, 76 ± 13, and 70 ± 12% of TNF-α-induced levels by 4R treatment at concentrations of 4, 8, and 16 μM, respectively (Figure 5E).

The adhesion of circulating monocytes to endothelial cells is an early step in inflammation. The monocyte adhesion assays with U-937 cells demonstrated the anti-inflammatory effects of 4R at the functional level. As shown in Figure 5F, when endothelial cells were stimulated by TNF-α, the percentage of U937 cell adhesion to endothelial cells was increased to 26 ± 0.021% compared to that in non-activated endothelial cells with 17 ± 0.01% adherent monocytes. 4R treatment reduced the percentage of adherent U937 cells in a dose-dependent manner. The percentage of the adherent cells was 22 ± 0.006, 18 ± 0.002, 13 ± 0.006% in 4, 8, and 16 μM 4R treatments, respectively (Figure 5F). The results shown in Figures 5D–F indicate that 4R has anti-inflammatory activity, which most likely will have a beneficial effect on PD outcomes.

## Discussion

**Figure 6:**
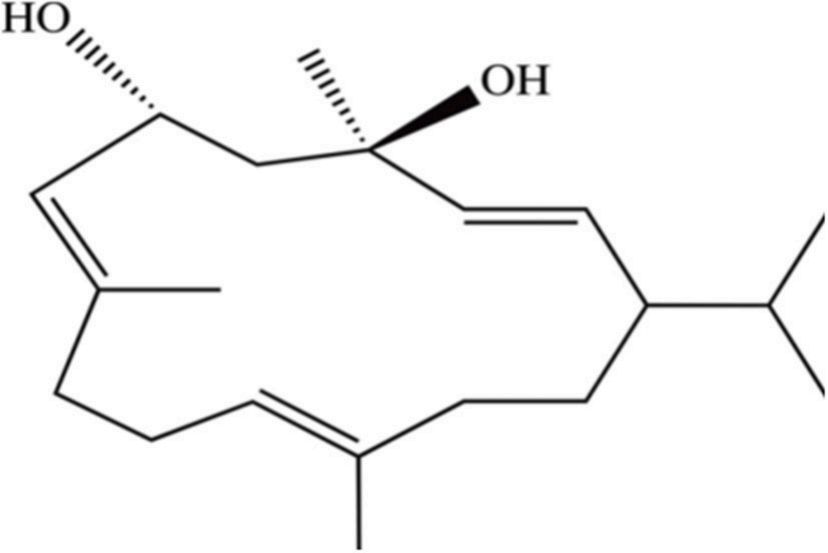
**The structure of 4R**.

Some studies have indicated that exposure to tobacco reduces the risk of PD (Hernan et al., 2002; Gale and Martyn, 2003; Castagnoli and Murugesan, 2004; Chen et al., 2010; De Palma et al., 2010). One of the explanations for this observation is that nicotine exposure can promote dopaminergic neuron survival. However, there are many other compounds in tobacco leaves which could contribute to this protective effect. Cembranoids seem to be likely candidates for the neuroprotection exerted by tobacco. Cembranoid content in green leaves of tobacco is about 1% (w/w) which is similar to the content of nicotine (1–3%) (Hann et al., 1998; Ferchmin et al., 2001, 2009). 4R is the second most abundant cembranoid in tobacco plants. It is a stable, lipophilic small molecule (MW: 306) (Figure 6) that easily passes through the BBB reaching the brain. The concentration of 4R in the brain is higher than that of the plasma and remains in the brain for several hours (Velez-Carrasco et al., 2015). Brain-permeability of 4R was also evidenced by blockage of the central effect on behavioral sensitization to nicotine and inhibition of neuronal acetylcholine receptor (nAchR) function by intraperitoneal injection of 4R in rats (Ferchmin et al., 2001). The neuroprotective activity of 4R in hippocampal slices was shown to be mediated by the interaction between α7 and α4β2 nAChRs followed by activation of the PI3-kinase/Akt anti-apoptotic cascade (Ferchmin et al., 2005, 2015). Furthermore, 4R was found to decrease the infarct size in mice and rats subjected to brain ischemia via inhibition of ICAM-1 expression and restoration of Akt phosphorylation (Martins et al., 2015).

The present results extend our previous findings regarding the neuroprotective effects of 4R. We found that 4R significantly attenuated TH reduction induced by 6-OHDA in both the striatum and SN on the lesioned side of the animals (Figure 2). The loss of dopaminergic neurons in the SN and of TH immunostaining in the striatum was reflected by corresponding functional deficits. The animals in the control groups performed worse over time in the behavior tests, reflecting the progressive loss of dopaminergic neurons; however, their behaviors were significantly improved in 4R-treatment groups in which both cylinder test and corner test scores were maintained close to the baseline (Figure 1). Furthermore, 4R was found to protect differentiated neuro-2a cells from 6-OHDA challenge (Figure 4), and this protection was associated with restoration of anti-apoptotic protein p-Akt expression (Figure 5A). We also found neuroprotection of 4R in the neuro-2a cells was associated with enhancement of HAX-1 levels (Figure 5B). Besides effects of 4R on p-Akt and HAX-1 expression, the level of cleaved caspase-3 was reduced by 4R treatment (Figure 5C). The above observations indicate that neuroprotection of 4R was through maintaining and activating the anti-apoptotic proteins, Akt and HAX-1, which leads to inhibition of pro-apoptotic caspase-3 activity. In addition to neuroprotection, the anti-inflammatory effect of 4R may also contribute to its therapeutic effect in PD. As shown in Figures 5D–F, 4R suppressed the TNF-α-induced monocyte adhesion to murine brain-derived endothelial cells by inhibiting expression of nuclear p65 and the NF-κB downstream inflammatory cytokine, VCAM-1. Monocyte adhesion is a functional indicator of the inflammatory response *in vitro*, and we used this assay to evaluate the effect of 4R on inflammation. 4R significantly inhibited U937 cell adhesion to the murine brain-derived endothelial cells (Figure 5F). These results are consistent with previous studies indicating anti-inflammatory effects of cembranoids (Thao et al., 2014; Martins et al., 2015). Anti-inflammatory effects of 4R in brain-derived endothelial cells suggest that it may have anti-inflammatory effects in the BBB *in vivo*. The likely mechanism of the anti-inflammatory effects of 4R is via inhibition of NF-kB activity in the endothelial cells, which leads to a reduction of its downstream inflammatory cytokines, including VCAM-1 and ICAM-1. Therefore, the anti-inflammatory effect of 4R on brain endothelial cells may provide additional protection for neurons against injury induced by various insults like ischemia and 6-OHDA. Notably, because 4R can pass through the BBB and access the brain, 4R may also have similar effects on microglia, which could contribute to its therapeutic effect in PD. Further study will be needed to elucidate 4R's effect on microglia.

In summary, previous studies have found that 4R has neuroprotective effects against acute injury induced by N-methyl-D-aspartate (NMDA), ischemia or organophosphorus compounds (Ferchmin et al., 2005, 2014; Eterovic et al., 2011, 2013; Martins et al., 2015). Our current study found that 4R has therapeutic effects in the rat 6-OHDA progressive PD model, which is a chronic injury. The present results are consistent with the following conclusions: First, 4R has significant neuroprotective activity in both the rat 6-OHDA-induced PD model *in vivo* and 6-OHDA-induced injury in differentiated neuro-2a cells *in vitro*. The therapeutic effect in the rat PD model is evident both at morphological and behavioral levels. Second, 4R's neuroprotection is associated with activation of the anti-apoptotic proteins Akt and HAX-1, and inhibition of vascular inflammation by inhibition of NF-κB activity. However, it is not known at this point if activation of Akt and HAX-1 are the consequences of the anti-inflammatory effect of 4R or a direct effect of 4R on these two proteins. The present findings may also provide new insight regarding the reduced risk for PD among cigarette smokers, which may have benefited from 4R in the tobacco, whereas it was traditionally thought to be mediated only by nicotine. Although the present results demonstrate the therapeutic efficacy of 4R in a model of PD *in vivo* when the drug was administered just after injection of 6-OHDA, the duration of the therapeutic window remains to be determined in future experiments. Furthermore, it is necessary to perform additional mechanistic studies *in vivo* to elucidate the mechanism(s) of action of 4R's therapeutic effect in PD. A previous study has indicated that 4R has no toxicity at the high concentration of 30 mg/kg in rats and 4R easily passes through BBB (Velez-Carrasco et al., 2015). 4R, or one of its analogs, is likely to have an impact on PD therapy and open the door for novel applications of tobacco products for medical purposes. Accordingly, the present study may provide new lead compounds for the development of novel therapeutic agents for PD.

## Ethics statement

All protocols involving the use of live rats were revised and approved by the Institutional Animal Care and Use Committee of the James L. Winkle College of Pharmacy, University of Cincinnati.

## Author contributions

JHu: Performed experiments, data acquisition, analysis and interpretation of data. Drafted manuscript. AH: Assisted in establishing the PD model; assisted with the stereology; revised the manuscript. KBS: Participated in setting up PD model. Revised the manuscript. VE: Participated in data analysis and interpretation. Revised the manuscript. PF: Drafted and revised the manuscript. Contributed to analysis, and interpretation of data. Provided the 4R for the study. JHao: Designed the study, analyzed and interpreted the data. Drafted the manuscript; approved the final version to be published.

### Conflict of interest statement

The authors declare that the research was conducted in the absence of any commercial or financial relationships that could be construed as a potential conflict of interest.
